# Virtual Delivery of Parent Coaching Interventions in Early Childhood Mental Health: A Scoping Review

**DOI:** 10.1007/s10578-023-01597-8

**Published:** 2023-09-23

**Authors:** Catriona Hippman, Janet W. T. Mah, Megan MacFadden

**Affiliations:** BC Children’s Hospital, University of British Columbia (UBC), 4500 Oak Street, Vancouver, BC V6H 3N1 Canada

**Keywords:** Virtual, Early childhood mental health, Scoping review, Parent coaching, Behavioural interventions

## Abstract

Parent–coaching interventions positively impact child development. Virtual delivery of such interventions is supported by literature reviews and a practice guideline, however, none of these focused on children under age six. A scoping review of virtually-delivered parent-coaching interventions for disruptive behaviour, anxiety, and parent–child relationship concerns in children under age six was conducted between Dec. 15, 2020 and April 22, 2021. Iterative searches of the databases PubMed, CINAHL, and PsycINFO were complemented by reference list searches and clinician expert review (*N* = 1146). After relevance screening and duplicate removal, collaboratively-developed inclusion criteria were applied to records, followed by data extraction from eligible articles (*n* = 30). Most literature documented behavioural-based interventions targeting disruptive behaviour which were delivered individually, by therapists, to White, non-Hispanic parents. Evidence supports feasibility and efficacy of virtually-delivered parent-coaching interventions to improve child disruptive behaviour (strong), anxiety (moderate), and parent–child relationship (weak). There is a significant gap in the literature regarding the virtual delivery of attachment-based parent-coaching interventions. In sum, virtual parent coaching can be an efficacious approach for children under age six, particularly for behavioural challenges.

**Supplementary Information:**

The online version contains supplementary material available at 10.1007/s10578-023-01597-8.

## Introduction

Disruptive behaviour, anxiety, and strain in the parent–child relationship are the most common reasons for families with children under age six to be referred for mental health services [1]. Anxiety disorders and disruptive behavioural disorders are very common in children under the age of six, with prevalence rates close to 10% each, which is very similar to rates amongst older children [2, 3]. Early intervention has been shown to positively impact later socio-emotional functioning and success in the school environment [4]. Treatment options can include parent coaching interventions, parent–child dyadic therapies, child-focused therapies (e.g., play therapy), and less commonly, pharmacotherapy [5]. Given developmental needs and the primacy of the parent–child relationship for this population (i.e., those under the age of six), involving the parent(s) and/or caregivers is crucial to the success of any treatment plan to address early childhood anxiety disorders or disruptive behaviour disorders [6, 7].

Two theoretical models which most commonly underpin these early childhood mental health interventions are behaviourism and attachment theory [1]. Behavioural-based parenting interventions have a rich evidence base, particularly for the treatment of disruptive behaviour [8]. A model for behavioural parent training that was developed by Hanf [9, 10] has been foundational to most subsequent behavioural parent training interventions, including the Incredible Years [11], and Helping the Noncompliant Child [12, 13]. The Hanf model drew from family systems theory, social learning theory, and operant conditioning principles to coach parents in the skilled use of positive reinforcement and restricted use of aversive consequences. Parents are taught positive parenting skills, particularly use of parental attention (in the form of praise and child-centered play) and positive reinforcement (such as incentive rewards), to shape and increase appropriate behaviour (e.g., compliance) in their children. Parents are also taught how to limit inappropriate child behaviour through prevention strategies (e.g., environmental and instructional accommodations) and select, purposeful use of consequences (e.g., removal of privileges). Moderate effect sizes for improvements in child disruptive behaviour is a well-established outcome for behavioural-based parenting interventions amongst several meta-analyses, and many studies have shown effectiveness with this intervention approach across settings and populations [14–20]. Further details of some behavioural-based parenting interventions can be found in Box 1.

Attachment-based parenting interventions draw on elements of attachment theory as first developed by Bowlby [21] and Ainsworth [22]. It is held widely among contemporary experts that one of the most useful applications of attachment theory is in the development of therapeutic interventions to support young children and their caregivers [23]. Therapeutic targets of attachment-based parenting interventions generally fall within two domains. The first target seeks to enhance caregivers’ reflective functioning capacities. The second target seeks to promote caregiving behaviours associated with secure attachment. The scope of attachment-based interventions is quite varied, with some interventions focusing more explicitly on enhancing caregiver reflection and others focusing more explicitly on the promotion of sensitive caregiving behaviours. Attachment-based interventions share a common goal of creating a therapeutic milieu of safety for caregivers, which creates the opportunity for caregivers to reflect and develop new parenting skills.

Among those attachment-based interventions with the strongest evidence base are Child Parent Psychotherapy (CPP) [24, 25], Video-feedback to Promote Positive Parenting (VIPP)[26, 27], and Attachment and Biobehavioural Catch-Up (ABC) [28]. Circle of Security (COS), an intervention directly informed by attachment theory, also has an emerging evidence base [29]. Attachment-based interventions have been shown to increase attachment security, increase parental sensitivity and improve emotional regulation skills in children [30, 31]. Other outcomes noted in studies of attachment-based interventions include reductions in parenting stress and reduced rates of disruptive behaviours in children [32, 33]. Attachment-based interventions have been implemented successfully with families at higher risk for parent–child relational problems, such as adoptive families and families whose members have been exposed to significant adversity, such as poverty, domestic violence and other forms of trauma [30, 34]. Further details of some attachment-based parenting interventions can be found in Box 1.

There have been strong opinions and misconceptions between practitioners who are predominantly behavioural-based or attachment-based [35]. For instance, behavioural strategies can be portrayed as undermining attachment security, while attachment approaches can be seen as permissive. Rather than seeing these approaches as contradictory, our perspective aligns with a rich literature that views both these approaches as complementary [1, 36, 37]. Both types of interventions emphasize positive parenting with concepts that are similarly aligned. That is, the behavioural focus on positive attention and setting limits is consistent with the attachment focus of “being with” and “taking charge”, respectively. In fact, Troutman has provided a framework for coaching parents from an integrated behavioural- and attachment- based perspective [1], and there are interventions that are both behavioral-based and attachment-based. One such intervention with an extensive evidence base is Parent–Child Interaction Therapy [12, 20].

### The Gap in the Literature and Study Purpose

While historically, there has been an emphasis on the importance of delivering interventions in-person, tele-mental health services have become increasingly popular [38]. Virtual delivery of services increases access to underserved populations (e.g., rural communities), and reduces barriers for families (e.g., reduces travel and childcare demands). During the COVID-19 pandemic, there was a seismic shift across the board in healthcare from a default of in-person care to reliance on virtual care to enable safe care delivery. However, this shift was motivated by necessity rather than evidence. Now that health care services are transitioning back to in-person care delivery, health care teams are re-evaluating how to strike the balance between in-person and virtual care provision. An understanding of the existing evidence-base regarding virtual care delivery can help to inform this decision-making process.

Several reviews have shown the effectiveness of virtual delivery of mental health services for children and adolescents [39–41], including a practice guideline published by the American Telemedicine Association [42]. While these guidelines included some considerations specific to those age six and under, the research on which they were based was mostly with children and youth over age six. A recent scoping review reported on the nature and range of early childhood mental health interventions [6]. However, this review only included articles published before 2013 and the authors acknowledged there were a number of studies of interventions delivered online that have been published since then. The purpose of the current scoping review was to respond to this gap in the literature by mapping available evidence of virtually-delivered parent coaching interventions to promote early childhood mental health.

## Methods

A scoping review of the literature was conducted between Dec. 15, 2020 and April 22, 2021 according to published methodology [43–45]. Scoping reviews are well suited to comprehensively mapping out available research in emerging areas such as this in contrast to systematic reviews, which are ideal for answering targeted questions by appraising the quality of a fairly sophisticated evidence base and synthesizing the results [46]. As is typical for scoping reviews [43], our search strategy was iterative, meaning that, as we became more familiar with the literature, we repeated steps in the scoping review process using additional, more sensitive and specific, search terms in an effort to ensure that our scoping review was comprehensive in its coverage of the available evidence. In our first set of searches, we searched the databases PubMed, CINAHL, and PsycINFO using the terms: “telepsychiatry”; “telemental health”; “e-health”; “psych*”; “telehealth”; “telemedicine”; “videoconferencing”; “teleconferencing”; and “videoteleconferencing” (See Supplemental material for exact search strings). For all searches, the following filters were applied: “Human”, “English”, “infant/ preschool” (defined by PubMed and CINAHL as 1 month—5 years, and by PsycINFO as 2 months—5 years). No filter was applied for publication date, so all publications in those databases until April 22, 2021 were included.

The first author assessed the titles and abstracts of all articles identified in the databases for relevance. All authors collaboratively drafted the inclusion and exclusion criteria for determination of eligibility, but this was iteratively refined over the course of reviewing titles and abstracts of identified records (Table 1). As part of this refinement process, the second author reviewed the first author’s screening of the titles and abstracts of the first 100 records for categorization into eligible, ambiguous (needs full text review), and ineligible. An Excel spreadsheet was used to support tracking and categorization, and provided an audit trail. Once the inclusion and exclusion criteria had been solidified, the first author reviewed the rest of the titles and abstracts of all articles identified using the first set of search terms. The full list of potentially eligible articles (those categorized as either ‘ambiguous’ or ‘eligible’) was then reviewed by the second author (*n* = 129). The full articles were obtained for those publications that were judged by both authors to be relevant, or to require further information to determine whether they met inclusion criteria. Reference lists of all potentially relevant review articles that were identified in the searches were reviewed to identify any additional articles for inclusion which hadn’t been identified in the initial searches. Review strategy and preliminary results (28 eligible articles) were assessed by two clinical investigators in the field (at the authors’ home institution; one with expertise in behavioural-based interventions, one with expertise in attachment-based interventions) to identify potential areas/articles that were missing. Based on this expert review and the preliminary results, we conducted a second set of searches of the databases PubMed, CINAHL, and PsycINFO using the same filters, but with the addition of the terms: “attach*”; “behav*”; “online”; “virtual”; “digital”; “remote”; and "mental health". Again, the reference lists of all potentially relevant review articles that were identified in the searches or suggested by the clinical investigators were reviewed for additional eligible articles. Also based on a recommendation from the clinical investigators, the first author searched clinicaltrials.gov for any studies that were registered but for which we had not found published results.

**Table 1:** Final inclusion and exclusion criteria

	Inclusion criteria	Exclusion criteria
Population (Participant characteristics)		
Mean age of children	6 or younger	Older than 6
Reason for concern/referral	Disruptive behaviour (including oppositional defiant disorder, or attention deficit hyperactivity disorder) Emotional dysregulation (e.g., anxiety) Parent–child relationship	No disruptive behaviour, emotional dysregulation, or parent–child relationship concerns Focus of record on diagnosed genetic syndrome with impact on mental health / behaviour / capacity to engage in interventions
Concept and Context (Intervention characteristics)		
Content	Delivery of therapeutic intervention Behaviour management, emotion regulation, or parent–child relationship	Assessment / consultation / education only Functional communication training, or interventions targeting socio-communication or adaptive functioning
Target	Parents or parent–child dyads	Educators / clinicians
Mode of therapeutic intervention	Virtual / telephone	In-person

Only original research articles were included. There were nine records (23%) that otherwise met inclusion criteria, which were clinical practice resources (*n* = 3; 10%), commentaries *(n* = 2; 6.67%), study protocols (*n* = 2; 6.67%), a review and theoretical framework (2.6%), and a book chapter (2.6%)

Full texts of all potentially relevant articles were reviewed by the first author, with secondary review by the second author of any articles whose eligibility was ambiguous. The focus of the full-text screening for articles categorized as ‘ambiguous’ was to search for information relevant to the inclusion and exclusion criteria, when it was not available in the abstracts. For example, the most common detail missing from the abstract was the average age of children who participated. At this stage, the authors recognized that, while most articles identified in the searches that were targeting difficulties in the parent–child relationship were not delivered virtually, there were a subset of records describing such interventions using video feedback. The authors felt that video feedback could be well suited to virtual delivery, given the compatibility and efficacy of video-conferencing platforms in healthcare [47]. Thus, we agreed upon an exploratory sub-aim for the review. This exploratory sub-aim was to describe the use of video feedback for interventions targeting difficulties in the parent–child relationship. For this sub-aim, articles identified in the course of the search that reported the use of video feedback in the context of interventions directed towards the parent–child relationship were summarized descriptively, without data charting. When the final set of eligible records had been determined, the first author drafted the fields for charting the data, and then all three authors met to review the data charting form and process, as recommended [43, 44]. After incorporating feedback into the charting form, the first author charted five articles and then shared the completed forms with the co-authors for review and further feedback. After incorporating this round of feedback, the first author completed data charting for the remaining eligible records which presented original research. Results were collated and summarized descriptively.

## Results

**Fig. 1:**
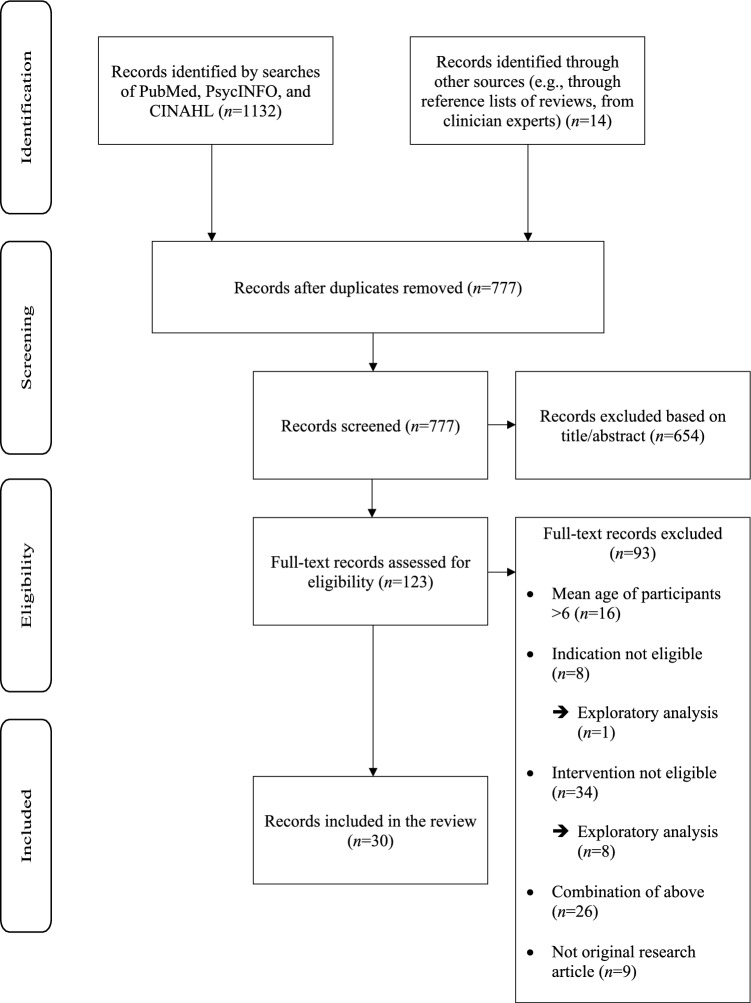
Flow diagram

A total of 1146 records were identified, and 777 were screened, following removal of duplicates. Figure 1 presents the process by which we identified eligible records for inclusion, with details about reasons for exclusion. A summary mapping out features of the available literature is presented in Table 2, detailed characteristics of all eligible original research articles included in the main analysis (*n* = 30) are presented in Table 3, and descriptions of adaptations of included interventions for virtual delivery are in Box 1.

**Table 2:** Summary of features of included articles (*n* = 30)

Feature	*n* (%)
Country of origin	
Australia	14 (46)
USA	7 (23)
Finland	3 (10)
Germany	2 (7)
Canada	2 (7)
New Zealand	2 (7)
Study design	
RCT	15 (50)
Supplemental analyses of RCTs^a^	5 (16)
Case study	3 (10)
Real-world implementation study	2 (7)
Pilot RCT	2 (7)
Feasibility study	1 (3.33)
Qualitative interviews	1 (3.33)
Mixed-methods study	1 (3.33)
Indication	
Disruptive behaviour	24 (80)
Emotional dysregulation (anxiety)	4 (13)
Parent–child relationship	2 (7)
Therapeutic intervention orientation	
Behavioural-based	22 (73)
Both behavioural- and attachment-based	6 (20)
Attachment-based	2 (7)
Therapeutic intervention format	
Individual	27 (90)
Hybrid—mix of group and individual	2 (7)
Group	1 (3)
Mode of therapeutic intervention	
Entirely virtual—to participant’s home/community location	15 (50)
Hybrid—virtual and by phone/in-person	8 (26.67)
By phone	6 (20)
Entirely virtual—clinic to clinic	1 (3.33)
Intensity of therapeutic intervention	
7 or fewer sessions	5 (16.67)
8—12 sessions	20 (66.66)
13 or more sessions	5 (16.67)
Therapist requirements	
Therapist-guided	24 (80)
Therapist—clinical / specialty training	15 (62.5)
Therapist – trained for the study	7 (29)
Therapist training unclear	2 (8.5)
Self-guided	6 (20)

^a^Long-term follow-up/mediator analysis/moderator analysis

*RCT* randomized controlled trial; *USA* United States of America

**Table 3:** Detailed characteristics of all eligible articles (*n* = 30), ordered within each category according to intervention name (alphabetical), study design (strongest to weakest), and finally in chronological order for those of the same intervention and study design

Intervention name	Study design	Year, Country, Citation	Sample characteristics	Mode of therapeutic intervention^1^	Intervention characteristics	Outcome measures	Outcomes
*Parent–child relationship*							
Child Parent Relationship Therapy (CPRT) (See Box 1)	Mixed-methods study	2017 USA [60]	*Size* N = 8 *Age* Eligible age range: 3—10 years, Mean age = 6, Participant age range = 3—10 years *Indication* parent–child relationship, Severity: community sample, self-referred *Special features* None	Entirely virtual – to participant’s home/ community location Group	10 sessions Therapist-guided Therapist – clinical/specialty training	*Quantitative* Porter Parental Acceptance Scale (PPAS) *Qualitative* Purpose-designed open-ended eight question online survey measuring parent perceptions of online CPRT	Quantitative *Study retention rate* 53.33% *Significant improvement* for parental acceptance of child’s feelings and needs (PPAS) Qualitative *Themes* Positive perceptions: (1) Convenience: working at their own pace; accessibility; no driving; from home; global access; (2) Sharing in the online hangout; (3) User experience (website); (4) Personal benefit Negative perceptions: (1) Technical difficulties; (2) Length of the group sessions (too short); (3) Virtual group interaction (inferior to one-to-one face-to-face interaction)
Emotional Attachment and Emotional Availability (EA2) online (See Box 1)	Pilot RCT	2015 USA [59]	*Size* N = 15 *Age* Eligible age range: 1.5—5 years, Mean age = 3.5, Participant age range = 1.92—5.17 *Indication* parent–child relationship, Severity: community sample, self-reported concerns *Special features* adoptive parents	Entirely virtual – to participant’s home/ community location Hybrid – mix of group and individual	6 sessions Therapist-guided Therapist – trained for the study	1. Emotional availability scales, fourth edition; 2. Emotional availability—Self report (EA-SR); 3. Emotional Attachment & Emotional Availability Clinical Screener (EA2-CS); 4. Attachment Q-Sort, Version 3.0; 5. Parenting Stress Index (PSI); 6. Child Behavior Checklist-Parent Report for ages 1.5 to 5 years (CBCL)	*Study retention rate* 100% *Significant improvements* for intervention group compared to delayed intervention group in: child behavioural problems (CBCL; *Cohen’s d* = 1.69), observed parent–child emotional availability (Emotional availability scales; *Cohen’s d* = 1.38–3.16), parental perceptions of emotional availability (EA-SR; *Cohen’s d* = 0.06–1.40), and observed parent–child emotional attachment (EA2-CS; *Cohen’s d* = 2.75) *No significant differences* by group reported for security (Attachment Q-sort) or stress (PSI)
*Anxiety*							
BRAVE-ONLINE (See Box 1)	RCT	2014 Australia [79]	*Size* N = 52 *Age* Eligible age range: 3–6 years, Mean age = 4.08 years, Participant age range: not reported *Indication* Anxiety, Severity: community sample, research diagnosis of social phobia, separation anxiety disorder, specific phobia or generalized anxiety disorder (Anxiety Disorders Interview Schedule for DSM-IV-Parent Version), with a clinical severity rating of at least 4 *Special features* None	Hybrid – virtual and by phone Individual	6 sessions Therapist-guided Therapist – trained for the study	1. Anxiety Disorders Interview Schedule for DSM-IV-Parent Version (ADIS-P), including a clinical severity rating (CSR) 2. Children's Global Assessment Scale (CGAS) 3. Preschool Anxiety Scale (PAS) 4. Child Behaviour Checklist—Internalizing subscale (CBCL-Int) 5. Purpose-designed satisfaction questionnaire (intervention group only)	*Study retention rate* 96% *Intervention engagement* increased over time (immediately post-intervention, 42.1% of parents had completed module 5 – the exposure session, while at 6 month follow-up, 73.9% of parents had completed module 5) *Satisfaction* Moderate/high *Significant improvements* for: severity of anxiety diagnosis (CSR; *η*^*2*^ = *0.176*), functioning (CGAS; *η*^*2*^ = *0.115*), and anxiety/internalizing symptoms (PAS; *η*^*2*^ = *0.131* & CBCL-Int; *η*^*2*^ = *0.152*) – both immediately post-intervention and at 6 month follow-up. Effect sizes provided are for group x time interaction immediately post-intervention *No significant differences* in percentage of children with anxiety diagnosis (remission rate; ADIS-P), number of anxiety diagnoses per child (ADIS-P)
Cool Little Kids Online (See Box 1)	RCT	2017 Australia [80]	*Size* N = 433 *Age* Eligible age range: 3–6 years, Mean age = 4.8 years, Participant age range: not reported *Indication* Anxiety, Severity: community sample, score > 30 on the Short Temperament Scale for Children (STSC) – Approach subscale (85%ile or greater) *Special features* None	Entirely virtual – to participant’s home/ community location Individual	8 sessions Self-guided	1. Revised Preschool Anxiety Scale (PAS-R) 2. Online Assessment of Preschool Anxiety (OAPA) 3. Strengths and Difficulties Questionnaire (SDQ) 4. Children’s Anxiety Life Interference Scale – Preschool Version (CALIS-PV) 5. Over-Involved/ Protective parenting scale (OI/P) 6. Purpose-designed satisfaction questionnaire (intervention group only)	*Study retention rate* 86.4% I*ntervention engagement* Mean number of modules accessed: 4 (out of 8) *Satisfaction* High *Significant improvements* for child anxiety symptoms (PAS-R; *Cohen’s d* = 0.38)—both immediately post-intervention and at 24-week follow-up, life-interference from child anxiety (CALIS-PV; *Cohen’s d* = 0.33-child, 0.35-family) – both immediately post-intervention and at 24-week follow-up, percentage of children with anxiety diagnosis (remission rate; OAPA; RR = 0.74, 95% CI = 0.58–0.94)—at 24-week follow-up *No significant differences* in child internalizing symptoms (SDQ), over-involved/protective parenting practices (OI/P)
Cool Little Kids Online	Above RCT – moderator analysis	2018 Australia [81]	As above	As above	As above	As above; Additional measures for moderator analysis: 1. Purpose-designed socio-demographic questionnaire 2. Kessler 10 Psychological Distress Scale (K10) 3. Program engagement: 3.1 Number of completed modules, 3.2 Number of logins to the online program, 3.3 Length of program use (number of days between first and last login), 3.4 Total time spent logged into the program, 3.5 Self-reported frequency of inter-module skills practice	The only time x group interaction identified as a significant predictor was access to a printer, with *access to a printer* significantly predicting lower anxiety scores at follow-up for the intervention group, but not the waitlist group Greater self-reported inter-module skills practice predicted lower child anxiety at follow-up, and access to a printer predicted greater inter-module skills practice
Internet-delivered Coaching Approach behaviour and Leading by Modeling (CALM) (I-CALM) (see Box 1, under I-PCIT)	Case study	2016 USA [49]	*Size* N = 1 *Age* Participant age = 6; Eligible age range: N/A, Mean age: N/A, Participant age range: N/A; *Indication* Anxiety, Severity: clinical—diagnosed with generalized anxiety disorder (CSR = 5) and separation anxiety disorder (CSR = 4) *Special features* Hispanic family	Entirely virtual – to participant’s home/ community location Individual	12 sessions Therapist-guided Therapist – clinical/specialty training	1. Anxiety Disorders Interview Schedule for the DSM-IV, Parent Version (ADIS-IV-P), including clinical severity ratings (CSR) 2. Fear Hierarchy ratings (FH) 3. Clinical Global Impression Scale (CGI) 4. Children’s Global Assessment of Functioning (CGAS) 5. Dyadic Parent–child Interaction Coding System-3rd Ed (DPICS-III) 6. Working Alliance Inventory (WAI)	*Satisfaction* very strong therapeutic alliance reported (WAI) *Significant improvements* for: child anxiety (FH and CGI ratings) – to the point that remission was achieved—patient did not meet criteria for anxiety disorders after treatment (ADIS-IV-P), overall functioning (CGAS scores), and positive parenting skills (DPICS-III)
*Disruptive behaviour*							
Helping the Non-compliant Child (HNC) (See Box 1)	Pilot RCT	2014 USA [50]	*Size* N = 22 *Age* Eligible age range: 3–8, Mean age = 5.67, Participant age range: 3–8; *Indication* Disruptive behaviour, Severity: community sample, scores within clinical range on the Eyberg Child Behaviour Inventory (ECBI)- Intensity or Severity subscales *Special features* low income families	Hybrid – in-person and virtual Individual	10 sessions; technology-enhanced version of HNC (TE-HNC): 8 sessions Therapist-guided Therapist – trained for the study	1. Eyberg Child Behaviour Inventory (ECBI)- Intensity and Problem subscales 2. Program engagement: 2.1 Percentage of completed sessions 2.2 Percentage of completed weekly calls 2.3 Percentage of completed daily skills practice 2.4 Percentage of completed assigned worksheets 3. Program start-up and implementation costs 4. HNC Consumer Satisfaction Scale 5. TE-HNC only: 5.1 Percentage of completed daily surveys, 5.2 Purpose-designed post-intervention questionnaire	*Study retention rate* 78.9% *Intervention engagement* Engagement higher for TE-HNC group (higher percentage of completed weekly sessions, weekly calls, and daily skills practice); TE-HNC group completed ~ 60% of daily surveys; positive feedback from the TE-HNC group regarding the impact of the technology enhancements on their partners' engagement in the program *Costs* Start-up (HNC)—$10/family, (TE-HNC)—$671/family Implementation cost to master a skill (HNC) – Mean = $82, (TE-HNC) – Mean = $80 *Satisfaction* mean score was higher for TE-HNC *Significant improvements* in child behavioural problem symptoms (ECBI-Intensity and Problem subscales for both groups). Magnitude of improvement was greater for TE-HNC compared to HNC (Between-group comparison: ECBI-Intensity *Cohen’s d* = 0.99, ECBI-Problem *Cohen’s d* = 0.54)
Internet-delivered Parent–Child Interaction Therapy (I-PCIT)	Implementation study—Effectiveness open trial	2021 Australia [61]	*Size* N = 27 *Age* Eligible age range: 1.5–4, Mean age = 3.02, Participant age range: not reported; *Indication* Disruptive behaviour, Severity: community sample, scores within clinical range on the Eyberg Child Behaviour Inventory (ECBI) *Special features* rural families	Entirely virtual – to participant’s home/ community location Individual	Mean: 13.2 sessions Therapist-guided Therapist – clinical/specialty training	1. Eyberg Child Behaviour Inventory (ECBI) 2. Dyadic Parent–Child Interaction Coding System, fourth edition (DPICS-IV) 3. Program engagement: 3.1 Percentage of participants completing program to mastery 3.2 Percentage of completed weekly homework sheets 4. Therapy Attitude Inventory (TAI)	*Study retention rate* 63% *Intervention engagement* Weekly homework sheet completion: 69% *Satisfaction* very high amongst those who completed the program: M = 44.7 (TAI) *Significant improvements* in: child behavioural problem symptoms (ECBI – intensity: *Cohen’s d* = -1.43, ECBI – problem: *Cohen’s d* = -1.05, DPICS-IV child compliance: *Cohen’s d* = 0.33), and parenting skills (DPICS-IV positive parenting behaviours increased: *Cohen’s d* = 0.96, and DPICS-IV negative parenting behaviours decreased: *Cohen’s d* = -0.93)
Internet-delivered Parent–Child Interaction Therapy (I-PCIT)	RCT	2017 USA [48]	*Size* N = 40 *Age* Eligible age range: 3–5, Mean age = 3.95, Participant age range: not reported; *Indication* Disruptive behaviour, Severity: clinical sample, diagnosed with ODD, CD, or DBD-NOS (K-DBDS, DSM-IV criteria) and scores within clinical range on the Eyberg Child Behaviour Inventory (ECBI) *Special features* none	In-person (comparison clinic-based PCIT group) or virtual (I-PCIT group) Individual	Mean: 21.2 sessions Therapist-guided Therapist – clinical/specialty training	1. Kiddie-Disruptive Behavior Disorders Schedule (K-DBDS) 2. Clinical Global Impression-Severity and Improvement Scales (CGI-S/I) 3. Children’s Global Assessment Scale (CGAS) 4. Eyberg Child Behaviour Inventory (ECBI) 5. Child Behaviour Checklist (CBCL) 6. Barriers to Treatment Participation Scale (BTPS) 7. Client Satisfaction Questionnaire (CSQ-8) 8. Therapy Attitude Inventory (TAI)	Feasibility *Study retention rate* 87.5% (to post-intervention time-point) *Barriers*- fewer barriers for I-PCIT compared to clinic-PCIT (BTPS) *Intervention engagement* 70% of families in both groups completed their full treatment course *Satisfaction*- high for both groups, no difference between groups (CSQ-8, TAI) I-PCIT M TAI = 45.9, PCIT M TAI = 45.1 Efficacy *Significant improvements* across time for both PCIT and I-PCIT in: child behavioural problem symptoms (ECBI-Intensity: PCIT—*Cohen’s d* = -2.40, I-PCIT—*Cohen’s d* = -1.92, ECBI-Problem: PCIT—*Cohen’s d* = -1.28, I-PCIT—*Cohen’s d* = -1.15, and CBCL-externalizing: PCIT—*Cohen’s d* = -0.59, I-PCIT—*Cohen’s d* = -1.10). There were no significant group x time interactions. Effect sizes provided are for pre-intervention to immediately post-intervention comparisons *No significant differences* in diagnostic response to treatment (K-DBDS) or global ratings of severity, impairment, and functioning (CGI-S/I, CGAS)
Internet-delivered Parent–Child Interaction Therapy (I-PCIT)	Longitudinal cohort (pre- and post-intervention qualitative interviews)	2020 Australia [50]	*Size* N = 9 (10 parents, 13 interviews) *Age* Eligible age range: 2–4, Mean age: not reported, Participant age range: not reported; *Indication* Disruptive behaviour, Severity: clinical sample, receiving I-PCIT through clinical service *Special features* rural families	Entirely virtual—to participant’s home/ community location Individual	Mean: Not reported Therapist-guided Therapist—clinical/specialty training	N/A (Thematic analysis, in an essentialist-realist theoretical framework)	*Pre-treatment themes* (1) motivation for seeking treatment, and 2) barriers to previous service access (geographic)—> *acceptability* *Post-treatment themes* (1) positive outcomes—> *effectiveness*; (2) valuable program components (notably strong therapeutic alliance)—> *acceptability and effectiveness*, and challenges—> *feasibility*; and 3) acceptability of internet delivery—> *feasibility and acceptability*
Internet-delivered Parent–Child Interaction Therapy-callous-unemotional trait adaptation (I-PCIT-CU; see Box 1, under I-PCIT)	Case study	2017 Australia [52]	*Size* N = 1 *Age* Participant age = 5.08; Eligible age range: N/A, Mean age: N/A, Participant age range: N/A; *Indication* Disruptive behaviour, Severity: clinical—diagnosed with ADHD, pre-treatment ECBI Intensity subscale T score of 61 (raw score = 134) *Special features* rural Caucasian family	Hybrid – in-person and virtual Individual	12 sessions Therapist-guided Therapist – clinical/specialty training	1. Eyberg Child Behaviour Inventory (ECBI; completed by parents) 2. Sutter-Eyberg Student Behavior Inventory–Revised (SESBI-R; completed by teacher) 3. Inventory of Callous- Unemotional Traits (ICU) 4. Dyadic Parent–Child Interaction Coding System, fourth edition (DPICS-IV)	*Significant improvements* in: child behavioural problem symptoms (ECBI-Intensity, ECBI-Problem, SESBI-R-Intensity), and levels of callousness and unemotionality (ICU) *Outcomes maintained* at 3 months follow-up
Internet-delivered Parent–Child Interaction Therapy (I-PCIT)	Case study	2020 Australia [85]	*Size* N = 2 *Age* Case 1 = 2.5, Case 2 = 3; Eligible age range: N/A, Mean age: N/A, Participant age range: N/A; *Indication* Disruptive behaviour, Severity: clinical sample, scores within clinical range on the Eyberg Child Behaviour Inventory (ECBI) *Special features* rural families (race/ethnicity not reported)	Entirely virtual—to participant’s home/ community location Individual	Mean numbers of sessions: Case 1 = 14, Case 2 = 20 Therapist-guided Therapist—clinical/specialty training	1. Eyberg Child Behaviour Inventory (ECBI) 2. Dyadic Parent–Child Interaction Coding System, fourth edition (DPICS-IV)	*Significant improvements* in child behavioural problem symptoms (ECBI-Intensity, ECBI-Problem) from clinical range at pre-treatment to non-clinical range at post-treatment, and parenting skills (DPICS-IV positive parenting behaviours increased)
Parenting Matters	RCT	2013 Canada [82]	*Size* N = 178 *Age* Eligible age range: 2—5 years, Mean age = 3.2, Participant age range: not reported *Indication* Disruptive behaviour, Severity: community sample, self-reported concerns *Special features* none	Entirely by phone Individual	6 sessions 6-section "Parenting Matters" self-help booklet, with telephone consults at weeks 0, 2, and 5 Therapist-guided Therapist – trained for the study	1. Eyberg Child Behaviour Inventory (ECBI) 2. Parenting Scale (PS) 3. Child Behaviour Checklist (CBCL) 4. Purpose-designed satisfaction questionnaire (intervention group only)	*Study retention rate* 91% *Satisfaction* High *Significant improvements* at 12 months follow-up for intervention group compared to usual care group in: child behavioural problem symptoms (ECBI, CBCL; *Cohen’s d* ≤ 0.15) *No significant differences* immediately post-intervention for child behavioural problems (ECBI, CBCL), and parenting skills (PS), and at 12 months follow-up for parenting skills (PS)
Promoting Engagement for ADHD Pre-Kindergartners (PEAK) (See Box 1)	RCT	2018 USA [57]	*Size* N = 47 *Age* Eligible age range: 3—5 years 11 months, Mean age = 4.43, Participant age range: not reported *Indication* Disruptive behaviour—at risk for ADHD, Severity: community sample, research diagnosis of ADHD (Barkley diagnostic interview, DSM-5 criteria), and scores > 90%ile on at least one subscale relevant to ADHD of the Conners Early Childhood Rating Scale (CERS) *Special features* none	Hybrid—in-person and virtual Hybrid—mix of group and individual	10 sessions Therapist-guided Therapist—trained for the study	1. Conners Early Childhood Rating Scale (CERS) 2. Parent stress index-short form (PSI-SF) 3. Purpose-designed knowledge questionnaire 4. Intervention groups only: 4.1 Percentage of completed sessions 4.2 Treatment session fidelity checklist (therapist) 4.3 Treatment implementation fidelity checklist (parent) 4.4 Intervention Rating Profile-15 (IRP-15)	Feasibility *Study retention rate* 89% *Intervention engagement* Therapist fidelity to the manual: 90–100% across cohorts; Parent treatment implementation fidelity: increased for both groups over time, with face-to-face group higher at mid-point, but not different post-intervention *Acceptability* scores in moderately acceptable range for both groups (IRP-15). Mean score for face-to-face group higher than for online group Efficacy *Significant improvements across time* in child behavioural problem symptoms (CERS-GI-Total: *η*^*2*^ = *0.19*), specifically restlessness/impulsivity and mood/affect (CERS-GI-RI: *η*^*2*^ = *0.23*, CERS-M/A: *η*^*2*^ = *0.24*), and parental knowledge (*η*^*2*^ = *0.43*)– for both intervention groups (no differences between intervention groups). *Cohen’s d* effect sizes for group differences between the intervention groups and the waitlist group were medium to large, ranging from 0.60 to 1.49 *No significant differences* in parental stress (PSI-SF), child inattention/overactivity (CERS-I/O), and child defiance/aggression (CERS-D/A)
Research Unit on Behavioral Interventions—Parent Training (RUBI-PT) (See Box 1)	Feasibility study	2018 USA [86]	*Size* N = 14 *Age* Eligible age range: 3—7 years 11 months, Mean age = 5.8, Participant age range: Not reported *Indications* ASD and disruptive behaviour, Severity: community sample, community diagnosis of ASD *Special features* none	Entirely virtual—clinic to clinic Individual	11 sessions Therapist-guided Therapist—trained for the study	1. Purpose-designed Treatment Fidelity Checklist (TFC) 2. Parent Treatment Adherence Scale (PTAS) 3. Parent Satisfaction Questionnaire (RUPP Autism Network) 4. Purpose-designed Telehealth Caregiver Satisfaction Survey 5. Purpose-designed Telehealth Provider Satisfaction Survey 6. Aberrant Behavior Checklist (ABC) 7. Home Situations Questionnaire—Autism Spectrum Disorder (HSQ-ASD) 8. Parent Target Problems (PTP) 9. Clinical Global Impression—Improvement scale (CGI-I) 10. Vineland Adaptive Behavior Scales—Second edition, Parent Interview Format (VABS-II)	Feasibility *Study retention rate* 92.9% *Intervention Engagement* Session attendance = 91.6%; Parent treatment implementation fidelity = 94.6% of in-session goals completed; Therapist fidelity to the manual = 98.2% *Satisfaction* Parent satisfaction: 100% would recommend the program to other parents (including by telehealth); Provider satisfaction: all felt confident in delivering the program via telehealth, but only 50% reported feeling that delivery via telehealth was "just as good" as delivery in clinic (the other 50% were unsure) Preliminary efficacy *Significant improvements* for parent-reported child irritability (ABC-I: *Cohen’s d* = 1.25) and cooperative behaviour (HSQ-ASD: *Cohen’s d* = 0.86). Independent evaluator also rated 78.6% of the cases as "much improved" or "very much improved" (CGI-I) *No significant differences* in parent-reported child adaptive behaviour (e.g., socialization, communication, and daily living skills; VABS-II)
Strongest Families Smart Website—SFSW; see Box 1)	Implementation study	2019 Finland [64]	*Size* N = 882 *Age* Eligible age range: 4 years, Mean age = not reported, Participant age range: not reported *Indication* Disruptive behaviour, Severity: community sample, score ≥ 5 on the conduct subscale of the Strengths and Difficulties Questionnaire (SDQ) *Special features* none	Entirely virtual – to participant’s home/ community location Individual	11 sessions Therapist-guided Therapist–clinical/specialty training	1. Time spent on the SFSW 2. Percentage of primary SFSW screens visited 3. Duration of coaching phone calls 4. Purpose-designed program satisfaction and therapeutic alliance questionnaire	*Study retention rate* 87.6% [Attrition rate of 12.4% was half that of the RCT intervention group of [65], which had an attrition rate of 24.8%] *Intervention Engagement* Time spent on the SFSW: 452 min [more than RCT group, which spent 431 min]; Percentage of primary screens visited: not reported; Duration of coaching phone calls: 37–38 min [same as RCT group]; Mean number of coaching phone calls: 11 [more than RCT group, which had a mean of 10] *Satisfaction* High–for both website and coaches [same as for RCT group]
Strongest Families (see Box 1)	RCT	2011 Canada [87]^2^	*Size* N = 80 *Age* Eligible age range: 3—7 years, Mean age = 4.92, Participant age range: not reported *Indication* Disruptive behaviour, Severity: community sample, research diagnosis of ODD (mild/moderate; Schedule for Affective Disorders and Schizophrenia—Present and Lifetime Versions (K-SADS-PL)) *Special features* none	Entirely by phone Individual	12 sessions Therapist-guided Therapist – trained for the study	1. Schedule for Affective Disorders and Schizophrenia—Present and Lifetime Versions (K-SADS-PL) 2. IOWA Connors Rating Scale 3. Disruptive Behaviour Rating Scale–Revised 4. Purpose-designed satisfaction questionnaire (intervention group only)	*Study retention rate* 100% *Intervention engagement* Session = 89% Video = 83% Skill implementation completion = 80% *Satisfaction* High *Significant improvements* for child behavioural problems, with fewer having a diagnosis post-intervention in the intervention group compared to the usual care control group (K-SADS-PL, IOWA Connors Rating Scale, Disruptive Behaviour Rating Scale–Revised) – at both 120 days (OR = 4.35, CI = 1.41–13.46) and 240 days (OR = 2.93, CI = 1.04–8.20) post-intervention. At 365 days post-intervention, the difference was no longer statistically significant, but the control group was more than twice as likely to have a diagnosis than the intervention group
Strongest Families Smart Website (SFSW)	RCT	2016 Finland [65]	*Size* N = 464 *Age* Eligible age range: 4 years, Mean age = not reported, Participant age range: not reported *Indication* Disruptive behaviour, Severity: community sample, score ≥ 5 on the conduct subscale of the Strengths and Difficulties Questionnaire (SDQ) *Special features* none	Entirely virtual—to participant’s home/ community location Individual	11 sessions Therapist-guided Therapist—clinical/specialty training	1. Child Behaviour Checklist (CBCL 1.5–5) 2. Parenting Scale (PS) 3. Parent Problem Checklist (PPC) 4. Sense of Coherence Scale (SOC-13) 5. Depression Anxiety and Stress Scale Short Form (DASS-21) 6. Inventory of Callous-Unemotional Traits (ICU) 7. Purpose-designed program satisfaction and therapeutic alliance questionnaire (intervention group only)	*Study retention rate*: 75.2% *Satisfaction*: High (84–98%) *Significant improvements* over time compared to the educational control group for: child behavioural problem symptoms (CBCL—externalizing subscale –at 6 and 12 months post-intervention: *Cohen’s d* = 0.34; CBCL—total score: *Cohen’s d* = 0.37 and internalizing subscale: *Cohen’s d* = 0.35 – at 12 months; ICU-Total – at 6 months: *Cohen’s d* = 0.14), and parenting skills (PS-Total – at 6 and 12 months: *Cohen’s d* = 0.53). Effect sizes provided are for the comparison between pre-intervention and 12 months post-intervention *No significant differences* over time by group for parental depression/ anxiety/ stress (DASS-21)
Strongest Families Smart Website (SFSW)	Above RCT—moderator analysis	2018 Finland [88]	As above	As above	As above	As above; additional measure for moderator analysis: 1. The Barkley Adult ADHD Rating Scale–IV (BAARS-IV-Quick Screen)	There was no moderating effect of parental ADHD (BAARS-IV-Quick Screen) or parental distress (DASS-21) on child behavioural problem symptoms (CBCL-externalizing) or parenting skills (PS)
Triple P (See Box 1)	RCT	1997 Australia [51]	*Size* N = 24 *Age* Eligible age range: 2—6 years, Mean age = 4.27, Participant age range: not reported *Indication* Disruptive behaviour, Severity: community sample, self-reported concerns and scores within clinical range on the Eyberg Child Behaviour Inventory (ECBI) *Special features* rural families	Entirely by phone Individual	10 sessions 10-section book and accompanying workbook (Level 1 Triple P materials), with brief weekly telephone consults (mean length = 20 min) Therapist-guided No details provided regarding the "therapists" supporting the intervention	1. Eyberg Child Behaviour Inventory (ECBI) 2. Parenting Sense of Competence (PSOC) 3. Parenting Scale (PS) 4. Depression Anxiety and Stress Scale (DASS) 5. Parent Daily Report Checklist (PDRC) 6. Purpose-designed consumer satisfaction inventory (intervention group only)	*Study retention rate* 100% *Satisfaction* High *Significant improvements* over time compared to the waitlist group for: child behavioural problem symptoms (ECBI; PDRC), parental self-esteem and efficacy (PSOC), parenting skills (PS), and parental distress (DASS). Effect sizes were not reported Percentage of children with ECBI scores above clinical cut-off (score of 127) at post-treatment: Waitlist group = 100% Intervention group = 33% *Outcomes maintained* at 4 months follow-up
Triple P	RCT	2006 Australia [54]	*Size* N = 41 *Age* Eligible age range: 2—6 years, Mean age = 3.91, Participant age range: not reported *Indication* Disruptive behaviour, Severity: community sample, self-reported concerns and scores within elevated range on the Eyberg Child Behaviour Inventory (ECBI; Intensity score ≥ 127 or Problem score ≥ 11) *Special features* rural families	Entirely by phone Individual	10 sessions 10-section book and accompanying workbook (Level 1 Triple P materials), with brief weekly telephone consults (mean length = 20 min) for those in the “enhanced self-directed” group Hybrid – mix of therapist-guided and self-guided (depending on group assignment) Therapist – clinical/specialty training	1. Eyberg Child Behavior Inventory (ECBI) 2. Parent Daily Report (PDR) 3. Parenting Scale (PS) 4. Parenting Sense of Competency Scale (PSOC) 5. Parent Problem Checklist (PPC) 6. Depression Anxiety Stress Scales (DASS) 7. Client Satisfaction Questionnaire (CSQ; intervention groups only)	*Study retention rate* 98% *Satisfaction* High (for both intervention groups, but higher for enhanced self-directed group; CSQ) *Significant improvements* over time compared to the waitlist group for: child behavioural problem symptoms (ECBI; PDR—both intervention groups), parental self-esteem and efficacy (PSOC—enhanced self-directed group only), parenting skills (PS—enhanced self-directed group only). Effect sizes were not reported Reliable Change Index (using ECBI Intensity scores): Enhanced self-directed group = 69% Self-directed group = 60% Waitlist = 0% *No significant differences* over time by group for inter-parental conflict over parenting (PPC) and parental distress (DASS) *Outcomes maintained* at 6 months follow-up, with the exception of parenting skills (PS), which improved for the self-directed group, and deteriorated for the enhanced self-directed group, such that there was no longer a significant difference between the intervention groups (no data for waitlist group at 6 months follow-up) Reliable Change Index (using ECBI Intensity scores): Enhanced self-directed group = 69% Self-directed group = 57%
Triple P online (TPOL; see Box 1, under Triple P)	RCT	2012 Australia [62]	*Size* N = 116 *Age* Eligible age range: 2 – 9 years, Mean age = 4.7, Participant age range: not reported *Indication* Disruptive behaviour, Severity: community sample, self-reported concerns and scores within clinical range on the Eyberg Child Behaviour Inventory (ECBI) *Special features* none	Entirely virtual – to participant’s home/ community location Individual	8 sessions Self-guided	1. Eyberg Child Behavior Inventory (ECBI) 2. Strengths and Difficulties Questionnaire (SDQ) 3. Family Observation Schedule (FOS)—adapted version 4. Parenting Scale (PS) 5. Parenting Task Checklist (PTC) 6. Depression Anxiety Stress Scales (DASS-21) 7. Parental Anger Inventory (PAI) 8. Parent Problem Checklist (PPC) 9. Intervention group only: 9.1 Time spent on each module 9.2 Client Satisfaction Questionnaire (CSQ)	*Study retention rate* 92% *Intervention Engagement* Average program completion time = 5.9 h (range 2.3–13.2); average module completion time: 40–74 min (40 min—module 7; 74 min—modules 1 and 4); After 9 months of access to TPOL, 95% of participants had completed module 1, and 47% had completed module 8 *Satisfaction* High (CSQ) *Significant improvements* over time compared to the waitlist group for: parent-rated child behavioural problem symptoms (ECBI-Intensity: *Cohen’s d* = 0.89, ECBI-Problem: *Cohen’s d* = 0.71; SDQ- Emotion: *Cohen’s d* = 0.44 and Conduct *Cohen’s d* = 0.58 subscales), parenting skills (PS: *Cohen’s d* = 0.53–0.61 for the three subscales), parental confidence in managing difficult child behaviour (PTC-Behavior: *Cohen’s d* = 0.84, PTC-Setting: *Cohen’s d* = 0.64), and parental anger in response to difficult child behaviour (PAI-Problem: *Cohen’s d* = 0.27, PAI-Intensity: *Cohen’s d* =) 0.29 *No significant differences* over time by group for: researcher-rated child behavioural problem symptoms (FOS), inter-parental conflict over parenting (PPC), and parental distress (DASS) *Outcomes maintained* at 6 months follow-up, with the exception of parent-rated child behavioural problem symptoms assessed by the SDQ (treatment gains maintained according to the ECBI). Further, *significant improvements* noted at this timepoint for the TPOL group relative to waitlist group for: researcher-rated child behavioural problem symptoms (FOS: *Cohen’s d* = 0.14), parental stress (DASS-stress: *Cohen’s d* = 0.59), and inter-parental conflict over parenting (PPC-Problem: *Cohen’s d* = 0.36, PPC-Extent: *Cohen’s d* = 0.33)
Triple P online (TPOL) versus Triple P hard-copy workbook	RCT (non-inferiority)	2014 New Zealand [55]	*Size* N = 193 *Age* Eligible age range: 3 – 8 years, Mean age = 5.63, Participant age range: not reported *Indication* Disruptive behaviour, Severity: "displaying elevated levels of disruptive behaviour problems"—no further details provided *Special features* none	Entirely virtual – to participant’s home/ community location Individual	8 sessions (TPOL) 10 sessions (hard-copy workbook) Self-guided	1. Eyberg Child Behavior Inventory (ECBI) 2. Parenting Scale (PS) 3. Parenting Task Checklist (PTC) 4. Parent–child relationship quality (PCRQ) 5. Child Abuse Potential inventory- brief version (Brief CAP) 6. Parental Anger Inventory (PAI) 7. Depression Anxiety Stress Scales (DASS-21) 8. Parent Problem Checklist (PPC) 9. Relationship Quality Inventory (RQI) 10. Client Satisfaction Questionnaire (CSQ)	*Study retention rate* 91.7% (to post-intervention time-point) *Non-inferiority analysis* TPOL demonstrated non-inferiority to the workbook group; with an effect size for the difference between groups being less than the chosen cut-off of 0.2 for impact on both child behavioural problem symptoms (ECBI: *Cohen’s d* ranged between -0.09 to -0.16), and parenting skills (PS: *Cohen’s d* ranged between -0.03 to 0.05) *Satisfaction* No difference between groups (CSQ, males and females)
Triple P online—brief (TPOL-brief; see Box 1, under Triple P)	RCT	2017 Australia [63]	*Size* N = 200 *Age* Eligible age range: 2—9 years, Mean age = 4.4, Participant age range: not reported *Indication* Disruptive behaviour, Severity: community sample, self-reported concerns and scores within clinical range (≥ 14) on the Strengths and Difficulties Questionnaire (SDQ) *Special features* none	Entirely virtual – to participant’s home/ community location Individual	5 sessions Self-guided	1. Eyberg Child Behavior Inventory (ECBI) 2. Child Adjustment and Parent Efficacy Scale (CAPES) 3. Parenting Scale (PS) 4. Purpose-designed Behavior Concerns and Parent Confidence Scale (BCPC) 5. Parent–Child Play Task Observation System (PCPTOS) 6. Depression Anxiety Stress Scales (DASS-21) 7. Parent Problem Checklist (PPC) 8. Parental Anger Inventory (PAI) 9. Intervention group only: 9.1 Number of completed modules 9.2 Percentage of completed pages and activities 9.3 Percentage completing a minimum dose of the introductory module plus one other module 9.4 Purposed-designed intervention evaluation survey 9.5 Client Satisfaction Questionnaire (CSQ)	*Study retention rate* 92.5% (to post-intervention time-point) *Intervention Engagement* Average number of completed modules = 2.74, Percentage completing a “minimum dose” = 62%, Percentage completing all 5 modules = 40%, average module completion time: 120 min – introductory module, 45 min – subsequent modules, Average number of logins = 6, Average total time spent on the program = 228 min *Satisfaction* High (Evaluation survey, CSQ) *Significant improvements* over time compared to the waitlist group for: parent confidence in managing difficult child behaviour (BCPC-Parent confidence: *Cohen’s d* = 0.45), and parenting skills (PS-Laxness: *Cohen’s d* = 0.26, PS-Over-reactivity: *Cohen’s d* = 0.24, PS-Verbosity: *Cohen’s d* = 0.55) *No significant differences* over time by group for: parent-rated child behavioural problem symptoms (ECBI, CAPES), child emotional problem symptoms (CAPES), parenting confidence in managing difficult child behaviour and emotions (CAPES), researcher-rated child behavioural problem symptoms. (PCPTOS), researcher-rated parenting skills (PCPTOS), parental distress (DASS-21), inter-parental conflict over parenting (PPC), and parental anger in response to difficult child behaviour (PAI) *Outcomes maintained* at 9 months follow-up Further *significant improvements* noted at this timepoint for the TPOL-Brief group relative to waitlist group for: parent-rated child behavioural problem symptoms (ECBI-Intensity: *Cohen’s d* = 0.41, ECBI-Problem: *Cohen’s d* = 0.27, BCPC-Behavior concerns: *Cohen’s d* = 0.39), and parenting confidence in managing difficult child behaviour and emotions (CAPES: *Cohen’s d* = 0.34)
Triple P online—Brief	Above RCT—moderator analysis	2017 Australia [89]	*Size* N = 100 (intervention group only) *Age* Eligible age range: 2—9 years, Mean age = 4.57, Participant age range: not reported Otherwise as above	As above	As above	As above	*Predictors of change in child behaviour* (ECBI) higher baseline child behavioural problem symptoms (ECBI), higher baseline inter-parental conflict over parenting (PPC), and greater parent age *Predictors of change in parenting skills* (PS) higher baseline parenting skills (PS) *Predictors of intervention engagement* (completing a minimum dose of the intervention): younger child age and lower baseline inter-parental conflict over parenting (PPC)
Triple P online (TPOL)	RCT	2018 Australia [56]	*Size* N = 183 *Age* Eligible age range: 2—8 years, Mean age = 3.5, Participant age range: 1—8 years Indication: Disruptive behaviour, Severity: community sample, self-reported concerns Special features: none	Hybrid—virtual and by phone Individual	8 sessions Hybrid—mix of therapist-guided and self-guided (depending on group assignment) Therapist—trained for the study	1. Eyberg Child Behavior Inventory (ECBI) 2. Parenting Scale (PS) Secondary outcome measures: 3. Depression Anxiety Stress Scales (DASS-21) 4. Parenting Tasks Checklist (PTC) 5. Parent Problem Checklist (PPC) 6. Relationship Quality Index (RQI) 7. Parental Anger Inventory (PAI) 8. Client Satisfaction Questionnaire (CSQ; intervention groups only)	*Study retention rate* 78.1% (to post-intervention time-point) *Intervention Engagement* *Program completion rate:*—Self-directed TPOL (TPOL-SD): 22.8% Practitioner-supported TPOL (TPOL-PS): 47% Average number of completed modules = 4.52 (out of 8), TPOL-PS group completed more modules (M = 5.62) than TPOL-SD group (M = 3.25), average module completion time: 62.95 min, average number of practitioner support phone sessions (TPOL-PS group) = 4.36, average call length: 23.69 min *Satisfaction* High (for both intervention groups, but higher for TPOL-PS group; CSQ) *Significant improvements* over time for both intervention groups compared to the waitlist group for: parent-rated number of child behavioural problems (ECBI-Problem – TPOL-SD *Cohen’s d* = 0.66, TPOL-PS *Cohen’s d* = 0.93), parenting skills (PS-Total – TPOL-SD *Cohen’s d* = 0.39, TPOL-PS *Cohen’s d* = 0.73), parental stress (DASS-21-Stress), parental confidence in managing difficult child behaviour (PTC), inter-parental conflict over parenting (PPC), and parental anger in response to difficult child behaviour (PAI). Effect sizes for significant secondary outcomes ranged between *Cohen’s d* = 0.32 and 0.57 for TPOL-SD and *Cohen’s d* = 0.34 and 0.57 for TPOL-PS Further *significant improvements* noted at this timepoint for the TPOL-PS group relative to waitlist group for: parent-rated frequency of child behavioural problems (ECBI-Intensity: *Cohen’s d* = 0.76), and parental depression and anxiety (DASS-21-Depression, DASS-21-Anxiety). Specific effect sizes not reported for secondary outcomes *No significant differences* over time by group for: parental relationship satisfaction (RQI) *Outcomes maintained* at 5 months follow-up, with the exceptions of parental stress (DASS-21-Stress) and parental anger in response to difficult child behaviour (PAI) for the TPOL-SD group Further *significant improvements* noted at this timepoint for the TPOL-SD group relative to waitlist group for: parental depression (DASS-21-Depression)
Triple P online (TPOL)	Above RCT—mediator analysis	2017 Australia [90]	As above	As above	As above	As above	*Significant mediating factors for parenting skills (PS)* parental confidence in managing difficult child behaviour (PTC) (for TPOL-PS but not TPOL-SD), such that higher levels of post-intervention confidence were associated with lower levels of negative parenting practices; greater intervention engagement (number of modules completed and number of phone sessions) was also associated with lower levels of negative parenting practices, partially through an association between intervention engagement and parental confidence (PTC) *Predictors of post-intervention parental confidence in managing difficult child behaviour (PTC)* Baseline levels of parental distress (DASS-Composite) (for TPOL-SD, but not TPOL-PS), with low levels of baseline distress associated with significant gains in parental confidence post-intervention (not true for high levels of baseline distress). This relationship between baseline distress and post-intervention parental confidence was not explained by intervention engagement (number of modules completed)
Triple P online (TPOL)	RCT	2020 New Zealand [53]	*Size* N = 53 *Age* Eligible age range: 3—4 years, Mean age = 4, Participant age range: not reported *Indication* Disruptive behaviour—ADHD, Severity: community sample, self-reported concerns and scores ≥ 14 on the Werry–Weiss–Peters (WWP) activity rating scale, and ≥ 16 on the Parental Account of Child Symptoms scale (PACS) *Special features* none	Hybrid – virtual and by phone Individual	8 sessions Therapist-guided Therapist – trained for the study	1. Conners Early Childhood Behavior Scale (Conners EC-BEH) 2. Child Behavior Scale (CBS) 3. Strengths and Difficulties Questionnaire (SDQ) 4. Parenting Scale (PS) 5. Authoritative Parenting scale of the Parenting Styles and Dimensions Questionnaire (PSDQ) 6. Depression Anxiety Stress Scales (DASS-21) 7. Parenting Sense of Competence scale (PSOC) 8. Client Satisfaction Questionnaire (CSQ; intervention group only)	Study retention rate: 86.8% (to post-intervention time-point) *Intervention Engagement* *Program completion rate* 55% 96% of participants completed module 1, and 55% completed module 8 *Satisfaction* High (CSQ) *Significant improvements* over time compared to the waitlist group for: parent-rated child behavioural problem symptoms (Conners EC-BEH-Hyp/Inatt: *Cohen’s d* = 0.52, Conners EC-BEH-Rest/Imp: *Cohen’s d* = 0.48, Conners EC-BEH-Def/Aggr *Cohen’s d* = 0.45), parent-rated child social functioning problems (Conners-EC-BEH-SocFunct: *Cohen’s d* = 0.47), teacher-rated child sociability (CBS-Prosocial subscale: *Cohen’s d* = 0.79), parenting skills (PS-Laxness: *Cohen’s d* = 0.64, PS-Over-reactivity: *Cohen’s d* = 1.11, PS-Verbosity: *Cohen’s d* = 0.63), positive parenting skills (PSDQ: *Cohen’s d* = 0.63), parental stress and depression (DASS-21-Stress: *Cohen’s d* = 0.76, DASS-21-Depression: *Cohen’s d* = 0.51), and parental sense of competence (PSOC-Satisfaction: *Cohen’s d* = 1.02, PSOC-Self-efficacy: *Cohen’s d* = 1.54) *No significant differences* over time by group for: teacher-rated child behavioural problem symptoms (SDQ) *Outcomes maintained* at 6 months follow-up for parenting skills (although not for the PS-laxness subscale or the PSDQ), parental stress and depression, and parental sense of competence. No longer significant differences for child behavioral problem symptoms or child social functioning Further *significant improvement* noted at this timepoint for the TPOL group relative to waitlist group for: parental anxiety (DASS-21-Anxiety: *Cohen’s d* = 0.61)
Wackelpeter und Trotzkopf	RCT	2013 Germany [83]	*Size* N = 48 *Age* Eligible age range: 3–6, Mean age = 5.2, Participant age range: not reported; *Indication* Disruptive behaviour, Severity: community sample, scores between 75–85%ile on the externalizing scale of the Child Behavior Checklist (CBCL) *Special features* None	Entirely by phone Individual	11 sessions 11-chapter self-help book (Wackelpeter und Trotzkopf, [93]), with brief weekly telephone consults Therapist-guided Therapist – clinical/specialty training	German versions of: 1. Child Behavior Checklist-Parent Report for ages 1.5 to 5 years (CBCL) 2. The Rating Scale for ADHD (FBB-ADHD) 3. The Rating Scale for ODD and CD (FBB-SSV) 4. The Home Situation Questionnaire (HSQ) 5. Parent Practices Scale (PPS) 6. Parenting Scale (PS) 7. Problem Setting and Behavior Checklist (PSBC) 8. Depression Anxiety Stress Scales (DASS) 9. Questionnaire on Judging Parental Strains (QJPS) 10. Parent Problem Checklist (PPC) 11. Purpose-designed satisfaction questionnaire	*Study retention rate* 96% *Satisfaction* High *Significant improvements* for intervention group compared to waitlist group in: child behavioural problem symptoms (CBCL-externalizing: *Cohen’s d* = 1.22, CBCL-internalizing *Cohen’s d* = 1.01; FBB-ADHD: *Cohen’s d* = 0.79; FBB-SSV: *Cohen’s d* = 0.89; HSQ: *Cohen’s d* = 0.88), parenting skills and self-efficacy (PS: *Cohen’s d* = 0.92, PSBC: *Cohen’s d* = 1.27), and parenting-related strains (QJPS: *Cohen’s d* = 1.03) No significant differences by group reported for parenting skills (PPS), parental depression/ anxiety/ stress (DASS), or inter-parental conflict (PPC)
Wackelpeter und Trotzkopf	Above RCT – 1 year follow-up	2015 Germany [84]	*Size* N = 36 Otherwise as above	As above	As above	As above	*Outcomes maintained* at 1 year follow-up—no significant change in any outcome measure scores between post-intervention to 1 year follow-up

^1^Interventions that were “entirely virtual” occasionally required supplemental use of a phone or premature termination of a session due to technical difficulties

^2^For this study, only the results for the ODD group were eligible for inclusion in the scoping review; child participants in the ADHD and Anxiety groups were over the mean age of six (exclusion criterion of age)

*ASD* autism spectrum disorder, *RCT* randomized controlled trial, *USA* United States of America

The majority of eligible literature was published in the past five years (*n* = 24, 80%), and only three records (10%) were published over a decade ago. From a methodological perspective, included articles were mainly randomized controlled trials (RCT; *n* = 22, 73.33%) focused on intervention outcomes; there were very few studies that included qualitative methods or focused on intervention process. The vast majority of participants in eligible studies were White, with only three studies (10%) for which more than a third of participants were racially/ethnically diverse [48–50]. Eligible ages for the children included in the studies ranged from 1.5 to 10 years, but the majority of studies included only children 6 years or younger (*n* = 20; 66.67%). Less than a quarter (*n* = 7, 23.33%) of the studies included in this review presented results for mothers and fathers separately [49, 51–55]; mostly parents were considered altogether and the majority of participants were mothers. Further, most studies reported numbers of males/females for children receiving interventions, but few reported analyses of differences in outcome by sex of the child.

### Feasibility

There is strong evidence that behavioural-based parent-coaching interventions targeting disruptive behaviour or anxiety in the early childhood population can be delivered virtually. All studies that measured parent satisfaction reported moderate-high intervention satisfaction or acceptability. With regards to program completion, on average, three quarters of participants completed therapist-guided behavioural-based programs (76.9%), while only a third of participants completed self-guided behavioural-based programs (33.68%). For self-guided programs, most participants completed half the available modules. Intervention engagement and satisfaction were generally greater with increasing therapist involvement [50, 54, 56, 57]. The three attachment-informed parent-coaching interventions identified in this review demonstrated the feasibility of delivering the interventions online. The study of the Emotional Attachment and Emotional Availability (EA2) program reported a remarkable 100% completion rate (with individual make-up sessions conducted). Participant feedback from the Child Parent Relationship Therapy (CPRT) online program highlighted a number of positive themes including convenience, ability to work at one’s own pace, accessibility, and benefits of not having to travel and being able to participate from one’s own home. Studies of internet-delivered Parent–Child Interaction Therapy (I-PCIT) have reported completion rates of 60–70%, with qualitative feedback from participants noting strong therapeutic alliance and lower barriers to I-PCIT compared to PCIT delivered in clinic.

For interventions using videoconferencing technology, loss of connection/disruption to the intervention was noted, and this represented a more significant barrier for geographically remote regions with poorer internet connectivity. It was possible to overcome this barrier if programs had the financial resources to provide participants with high quality internet, as seen in one study that sent participants an internet ‘dongle’ to access highspeed internet for mobile devices [58]. Few studies compared virtual delivery of interventions to in-person delivery, but participants in one such study noted fewer barriers to engaging in the intervention when delivered virtually [48], and this finding is echoed in the one qualitative study included in this review [58].

Only one study reported a cost analysis [50], and found a significantly higher start-up cost to delivering the intervention virtually (Technology-enhanced Helping the Noncompliant Child (TE-HNC)—$671/family) compared to treatment as usual (in clinic HNC—$10/family). It is important to note that the cost of the TE-HNC intervention included providing each family with a smartphone, including service plan, and tripod for use in video recording home practice sessions. Given the increasingly ubiquitous presence of smartphones in households, and decreasing costs of service plans, these start-up costs would be significantly reduced in the current (and likely future) technology climate. Interestingly, the implementation costs of mastering a skill in TE-HNC were slightly less (M = $80) compared to in-clinic HNC (M = $82). The costs of TE-HNC were lower because families in this group were able to master skills and complete the program more quickly, with a mean of eight sessions compared to an average of 10 sessions for the in-clinic HNC group. Thus, even with the greater intensity of therapist engagement in the TE-HNC group, therapists spent less time on this group overall, resulting in a lower implementation cost.

### Efficacy

For parent–child relationship concerns, virtual delivery of the attachment-informed intervention EA2 demonstrated efficacy in improving parent–child attachment and child problem behaviour [59], and an online version of CPRT demonstrated positive outcomes in the domain of parental acceptance of their children [60], all with large effect sizes.

Efficacy in improving child anxiety symptoms was demonstrated for the virtual delivery of the behavioural-based interventions BRAVE Online and Cool Little Kids Online, and the behavioural- and attachment-based intervention Internet-delivered Coaching Approach behaviour and Leading by Modeling (I-CALM; an adaptation of I-PCIT) (although the evidence for I-CALM is weak—only one case study [49]). BRAVE-Online demonstrated medium effect sizes in improvements in severity and symptoms of child anxiety, and child functioning, while Cool Little Kids Online demonstrated slightly lower magnitudes of small—medium effect sizes in child anxiety symptoms and child functioning. Remission rates for the two interventions were dramatically different post-intervention, with 60.1% for Cool Little Kids Online, compared to 34.8% for BRAVE-Online. However, at the 6-month follow-up assessment for BRAVE-Online, there was a remission rate of 70.6%, along with an increase in module completion rate (from 42.1% completion of module 5 (including the exposure session), to 73.9% completion of module 5).

Efficacy in improving child disruptive behaviour symptoms was demonstrated with at least one RCT, and generally large effect sizes, for the virtual delivery of the behavioural-based interventions Helping the Noncompliant Child (HNC), “Parenting Matters” (therapist-guided bibliotherapy), Promoting Engagement for ADHD Pre-Kindergartners (PEAK), Research Unit on Behavioral Interventions—Parent Training (RUBI-PT), Strengthening Families, Triple P, and “Wackelpeter und Trotzkopf” (therapist-guided bibliotherapy), and the behavioural- and attachment-based intervention I-PCIT. Only one study reported remission rate, which was a study of I-PCIT reporting a remission rate of 55% [48]. Effect sizes for intervention impact were larger, in general, as intensity of the intervention increased. This was demonstrated directly in some RCTs of interventions of differing intensity. For example, larger effect sizes were found for the group that had virtually delivered TE-HNC (which included smartphone enhancements like skills videos, daily progress surveys, mid-week video calls, and text reminders) compared to the group that had HNC in clinic [50]. Reliable and clinically significant change post-treatment in terms of child behaviour was demonstrated for the majority of participants for TE-HNC, I-PCIT, self-directed Triple P, enhanced self-directed Triple P, self-directed Triple P Online, and enhanced Triple P Online [48, 50, 51, 54, 61, 62].

Efficacy to enhance parenting skills was demonstrated, with generally medium—large effect sizes, for virtual delivery of the interventions I-PCIT, Strengthening Families, Triple P, and “Wackelpeter und Trotzkopf” (therapist-guided bibliotherapy). Evidence of efficacy for improving indirect parenting outcomes (e.g., parental distress or inter-parental conflict) was more inconsistent, but more likely with increasing intervention intensity. For example, no changes in distal parental outcome variables were found for a brief 5-session self-guided Triple P online program [63], whereas significant improvements in parental stress and inter-parental conflict were found for an 8-session Triple P online program, and further improvements in parental depression and anxiety were found when the Triple P online program had practitioner support [56].

### Effectiveness

This scoping review identified only two implementation studies—one for the Strongest Families Smart Website (SFSW) [64] and one for I-PCIT [61]. The implementation study of SFSW reported on the feasibility of its implementation in a real-world primary care context. It demonstrated higher engagement with the program (higher completion rate, time spent on the website, and number of coaching phone calls) relative to a comparison group in a previously published RCT [65], as well as similar levels of satisfaction with the website and coaches. It is important to note that ratings of child psychopathology were significantly higher in the implementation study, which could potentially explain the greater levels of program engagement for this group.

The implementation study of I-PCIT reported on both feasibility and effectiveness of its implementation in a real-world primary care context. It demonstrated similar levels of engagement and satisfaction to a comparison group in a previously published RCT [48]. In terms of effectiveness, 88.2% of those who completed treatment were below clinical cut-off at post-treatment assessment for frequency of disruptive behaviours (Eyberg Child Behavior Inventory-Intensity) and 82.4% were below cut-off for number of behaviours perceived by the family to be problematic (Eyberg Child Behavior Inventory-Problem), with very large and large effect sizes, respectively.

### Exploration of Parent Coaching Interventions Using Video Feedback to Improve the Parent–Child Relationship

Although this review only identified two studies of virtually-delivered parent coaching interventions that were solely attachment-based, and focused on parent–child relationship concerns, both interventions used video feedback to review parent–child interactions, which is a common therapeutic tool within attachment-based interventions [66]. The positive impact of video feedback in attachment-based interventions has been highlighted in two meta-analyses. The first meta-analysis found attachment-based interventions using video feedback to be more effective across outcomes than interventions that did not include a component of video-feedback [30]. The second meta-analysis, focusing specifically on family interventions that incorporate video feedback as a therapeutic tool, found video feedback to have a positive impact on parenting behaviour and parental attitude, as well as positive effects on the development of children [67]. One of the attachment-based interventions with the strongest evidence base is the Video-feedback Intervention to promote Positive Parenting (VIPP). This intervention relies on video-feedback, as does its various adaptations: VIPP-SD (Video-feedback Intervention to promote Positive Parenting and Sensitive Discipline) [68], VIPP-AUTI (Video-feedback Intervention to promote Positive Parenting adapted to Autism) [69], and VIPP-Co (Video-feedback Intervention for Co-parents of infants at risk for externalizing behaviour problems) [70]. Another video-feedback intervention with evidence from randomized controlled trials is AVI (Attachment-based Video Feedback Intervention) [71, 72].

Video-feedback has also been explored as an add-on to existing evidence-based interventions such as the Family Check Up [73]. In a preliminary study examining the effect of adding a video-feedback intervention component to the assessment feedback session of the Family Check-Up Intervention, researchers found that reviewing and engaging in feedback about videotaped interactions between parents and their children at age two predicted reduced caregivers’ negative relational schemas at age three, which acted as an intervening variable on the reduction of observed parent–child coercive interactions recorded at age five. Video-feedback has been applied successfully in interventions targeting populations with parents and children at risk for parent–child relational problems to promote attachment and reduce physiological dysregulation [66]. Further, it has shown potential in the context of cross-cultural healthcare delivery to racial or ethnic minority families [74]. When used intentionally, video feedback can act as a clinical engagement tool that can be used to enhance therapist cultural competence, and strengthen therapeutic alliance between clinicians and clients who come from different cultural backgrounds.

## Discussion

This scoping review mapped out literature regarding the virtual delivery of parent-coaching interventions for disruptive behaviour, anxiety, or parent–child relationship in the early childhood population. The vast majority of this literature has focused on interventions with a behavioural basis (with or without an additional foundation in attachment theory) targeting disruptive behaviour, delivered on an individual basis by therapists to White families. Although this review documented that the majority of publications investigating virtual delivery of parent coaching interventions were categorized as primarily behavioural- or attachment-based (*n* = 24, 80%), we advocate the benefits of both approaches for supporting families in the field of early childhood mental health.

There is consistent evidence supporting the feasibility of delivering parent-coaching interventions virtually, with high satisfaction and acceptability, and fewer barriers to access. Having said that, it is important to note the remaining potential for disparities in access for rural communities with limited internet infrastructure and other populations with lower resources, such as low-income families. In terms of efficacy, there is solid evidence that virtually-delivered behavioural-based interventions can improve child disruptive behaviour and parenting skills, with medium to large effects. These outcomes are in keeping with, or at times stronger than, available evidence supporting the delivery of these interventions in-person. Though effectiveness studies remain largely undocumented in the literature, there are two published studies that demonstrate successful implementation and strong outcomes in real-world primary care contexts of virtually-delivered interventions for child disruptive behaviour that are either behavioural-based or both behavioural- and attachment-based.

This review brings into focus the lack of published research on the efficacy of virtually-delivered interventions that are (1) solely attachment-based, and/or (2) focused on parent–child relationship concerns. That being said, although the pilot studies of EA2 online and CPRT online included in this review were limited by their small sample sizes and lack of randomization, both studies provide preliminary evidence of the efficacy of the virtual delivery of interventions targeting parent–child relationship concerns for caregivers and young children that are solely attachment- and/or relationship-based (not behavioural-based). They also highlight the potential benefits of video feedback in virtual care settings. Video feedback as a therapeutic tool within parent coaching interventions may be particularly amenable to virtual care delivery and is worthy of further study. For instance, there is a recent development of a virtually-delivered VIPP intervention [75].

### Implications for Practice

The global adoption of virtual health care during the COVID-19 pandemic has reinforced the benefits of virtually delivered interventions, which will likely continue to expand into the foreseeable future. For early childhood mental health interventions that focus on virtual coaching of parents and caregivers, there are several evidence-based programs that practitioners can consider. For the treatment of anxiety in the early childhood population, Cool Little Kids Online is an excellent option—it performed very well in terms of remission rate, and is an entirely self-guided intervention and so has a lower initial investment cost [76]. Minimal information was provided regarding characteristics of the populations studied, however, so further research is needed to explore the suitability of this intervention for different groups. Therapist involvement, for example, in the BRAVE-Online or I-CALM interventions, would likely be particularly important for tailoring treatment to the needs of specialized groups.

For the treatment of disruptive behaviour in the early childhood population, self-directed Triple P is a solid program [77]—either online (TPOL), or using the hard copy book and workbook, depending on internet availability and parent preference [55]. Reliable and clinically significant improvements in child disruptive behaviour have been demonstrated in multiple RCTs for parents using the self-directed Triple P intervention, which offers a lower therapist-time investment. The studies supporting the self-guided Triple P intervention were conducted with parents who were mainly White, highly educated, and of a moderate-high socio-economic status, although two studies did provide evidence in support of the efficacy of the intervention for parents who lived in rural areas, with lower levels of education, and of lower socio-economic status. Thus, therapist involvement in the interventions TE-HNC, I-PCIT, and enhanced Triple P (Online) would be key to tailoring treatment to be linguistically and culturally sensitive and appropriate for populations who are racialized or otherwise disadvantaged. Further, it is vital to consider and take steps to mitigate the risk of magnifying health inequities resulting from the digital divide, in which marginalized families are disproportionately excluded from virtually-delivered care [78].

### Limitations

This review was limited to articles published in English, so more evidence may exist in support of virtual delivery of these interventions in other languages, to more racially/ethnically diverse populations. Our use of the “infant/preschool” filters in the literature databases could have excluded some articles focused on children who were 6 years old (since the filters had an age limit of 5 years old). However, we did exclude 16 articles at the full-text review stage because their participants had a mean age greater than 6, so the filters did not screen out all research on populations over 5 years old. Additionally, we excluded articles that focused on providing interventions to parents of children with genetic syndromes or medical comorbidities that could impact engagement in the interventions; thus, we are unable to comment on the evidence base that may or may not exist in support of the feasibility, efficacy, or effectiveness of virtually delivering these interventions to these populations. As always, it is important to remain cognizant of the potential publication bias whereby studies with negative/non-significant results are more likely to be unpublished, thus biasing our findings towards intervention efficacy and effectiveness. Finally, given that this was a scoping review, rather than a systematic review, we did not evaluate the quality of the evidence or conduct an assessment of bias, and as such, this limits our capacity to draw conclusions in terms of practice recommendations or implications. The nature of the scoping review does, however, enable identification of gaps in the literature and recommendations for avenues that would be worthwhile for future research.

### Future Research

In particular, there is an urgent need for research to address the significant gaps in the literature identified by this review with respect to the virtual delivery of interventions for the early childhood population that are (1) solely attachment-based, and/or (2) focused on parent–child relationship concerns. Other potentially fruitful avenues of inquiry with currently limited literature bases for the early childhood population include the virtual delivery of: (1) group interventions, (2) interventions targeting anxiety, and (3) interventions to marginalized populations and rural populations. Future studies could also compare the “felt experience” of participants in virtually-delivered interventions versus in-person interventions, and whether this may impact intervention outcomes. It would also be useful for future research to include qualitative methods, studies of intervention process in addition to outcome, sex- and gender-based analyses, and to prioritize involvement of fathers/co-parents.

Finally, it would be worthwhile for future research to investigate the relative effectiveness, including cost-effectiveness, of different integrated care models. There is some preliminary evidence from this scoping review of the potential effectiveness of implementing a stepped-care model, in which patients first receive brief, low-intensity interventions, and initial non-responders “step up” to receive additional and/or more intensive treatment. This is supported by our scoping review given that: (1) brief online interventions that are entirely self-guided can improve outcomes and may be sufficient for a subset of the population in need, and (2) interventions of greater intensity have been demonstrated to have greater clinical impact. Future research could compare, for example, the unidirectional stepped care model versus a mechanism to screen referrals to triage those whose children have more severe symptoms into higher intensity interventions up-front (e.g., tiers of service), and the intersect between virtual versus in-person delivery of service at each stage or intensity level of intervention.

### Summary

In this scoping review of the literature regarding virtually-delivered parent-coaching interventions for disruptive behaviour, anxiety, or parent–child relationship in children under age 6, data were extracted from 30 eligible articles. The majority of these studies focused on interventions with a behavioural basis (with or without an additional foundation in attachment theory) targeting disruptive behaviour which were delivered individually, by therapists, to White, non-Hispanic parents. While evidence is somewhat limited, particularly with respect to the virtual delivery of solely attachment-based interventions and/or those targeting the parent–child relationship, the evidence that exists does support the efficacy of virtually delivering parent-coaching interventions to improve child disruptive behaviour, child anxiety, and the parent–child relationship for the early childhood mental health population. Avenues for future research in the area of virtual delivery of parent coaching interventions for the early childhood population identified by this review include: solely attachment-based interventions, interventions focused on the parent–child relationship or anxiety, delivery in a group format, inclusion of marginalized populations, rural populations, and fathers/co-parents, qualitative studies, studies focused on intervention process, sex- and gender-based analyses, and analyses of different integrated care models, including cost-effectiveness analyses.

**Box 1:** Descriptions of virtually-delivered parent coaching interventions

*BRAVE-ONLINE* (preschool version)—modified version of BRAVE-ONLINE (designed for children age 7–12); based on cognitive-behavioural therapy (CBT); 6 interactive module program (and two booster sessions) with written information, interactive games and quizzes, which are released sequentially following completion of each module; each module takes ~ 60 min to complete; this online program was supplemented by a booklet containing age-appropriate examples, explanations and applications for preschool-age children; in this study, an online therapist sent a follow-up email (manualized) after the completion of each module; telephone consult (15–30 min) provided at mid-point with parents' assigned online therapist; therapists were psychology graduate students, receiving weekly supervision from a registered psychologist
*Child Parent Relationship Therapy (CPRT) online*—based on filial therapy; a relationally-focused intervention; use of video feedback review as a core therapeutic technique; 10 week program with weekly sessions; includes asynchronous viewing of CPRT training video (45–60 min, content from CPRT treatment manual), synchronous group sessions (~ 45 min each), and video recordings of play sessions shared and discussed with the group; manualized—delivered in this study by a Master's-trained, licensed mental health counselor
*Cool Little Kids Online (CLKO)*—modified version of Cool Little Kids parenting group program; based on cognitive-behavioural therapy (CBT); 8 interactive module program with written information, videos, audio narration, interactive worksheets and activities (e.g., optional online diary for monitoring progress), and parent experiential stories, which are released weekly over 8 weeks; each module takes 30–60 min to complete, and parents are encouraged to practice skills and complete homework between modules; parents are guided through modules by an animated "coach"; telephone consult support with a psychologist was available when requested (was requested by only 5% of participants in this study)
*Emotional Attachment and Emotional Availability (EA2) online*—based on attachment theory, the emotional availability framework, and systems and transactional perspectives; 6 week program with weekly sessions, designed for 6–10 parents per session; includes bibliotherapy, video feedback (strengths-based) using an interactive website, and parent homework using a Parent Workbook; manualized—delivered in this study by a Master's-level graduate student under the supervision of a clinical and developmental psychologist
*Helping the Non-compliant Child (HNC)*—based on the Hanf model of behavioural parent training (Further details in (McMahon & Forehand, [13]). HNC materials are written at a 6th grade reading level. Length of HNC depends on time taken to achieve parental skill mastery, but is on average 8–12 sessions. HNC is manualized and was delivered in this study by Master's-level graduate students who participated in weekly supervision sessions - The technology-enhanced version of HNC (TE-HNC) included smartphone enhancements: (1) Skills video series; (2) Brief daily surveys; (3) Text message reminders; (4) Video recording home practice; and (5) Mid-week video calls
*Internet-delivered Parent–Child Interaction Therapy (I-PCIT)*—based on attachment and social learning theories, as well as developmental theories of parenting; includes in-session real-time feedback using a parent-worn Bluetooth earpiece device from a coach observing by videoteleconference (Further details in [91]). Length of I-PCIT depends on time taken to achieve parental skill mastery - In combination with Coaching Approach behaviour and Leading by Modeling (CALM) (I-CALM); PCIT-CALM is an adaptation of PCIT to target anxiety; it incorporates the "DADS" exposure scaffolding skills; in the case study included in this review, there were two co-therapists, who were both graduate students who had completed formal training in PCIT by a PCIT Master Trainer as well as advanced clinical training in exposure-based therapy for pediatric anxiety disorders - PCIT-CU is an adaptation of PCIT to accommodate needs specific to children with high callous-unemotional traits; it emphasizes a reward-based system—using a token economy as part of the procedure. In the case study included in this review, the family also received an adjunct treatment following I-PCIT-CU called Coaching and Rewarding Emotional Skills (CARES) to further target the callous-unemotional traits identified for this child. CARES involved 8 sessions, also conducted by videoteleconferencing, during which the child received training to recognize and respond to emotional cues (e.g., facial cues), complemented by emotion-based homework practice
*Research Unit on Behavioral Interventions—Parent Training (RUBI-PT)*—based on the Antecedent-Behaviour-Consequence model; manualized intervention with 11 core sessions and up to two supplemental sessions (60–90 min each) over 16 weeks, with homework assignments between sessions, and three telephone booster sessions up to 22 weeks; can be delivered by a range of providers, no specialized clinical designation required; training delivered by certified RUBI-PT therapist
*Promoting Engagement for ADHD Pre-Kindergartners (PEAK)*—theoretical basis was not specified; manualized intervention with 10 weekly sessions delivering behavioural parent training content (program outline in [57] Table 2, page S375) including lectures, brief quizzes, and videos; in this study, the online group was delivered individually, with weekly phone calls from a research assistant, and the face-to-face sessions were delivered in groups led by graduate students in psychology or special education, with supplemental group discussions and roles plays
*Strongest Families*—the "Parenting the Active Child" intervention (a component that targets children with ODD or ADHD within the larger Strongest Families program) was based on the Community Parent Education Program parent training approach previously described [92]. It includes 12 weekly sessions, and two booster sessions at 2 and 4 months post-intervention, with handbooks, videos, and weekly telephone coach sessions (mean length = 40 min); in this study, trained nonprofessional coaches delivered the intervention, supervised by a registered psychologist - The Strongest Families Smart Website (SFSW) delivers the Strongest Families program through an online platform which is interactive and personalized, with 11 weekly sessions, the option of two booster sessions ~ 2 and ~ 4 months post-intervention, and weekly 45 min phone calls conducted by a coach with healthcare professional certification, trained by an experienced Strongest Families clinician
*Triple P* (the Positive Parenting of Preschoolers program)—based on social learning principles, child and family behaviour therapy and applied behaviour analysis, social information-processing models, as well as developmental theories of parenting; it is comprised of 5 levels of increasing intensity, ranging from self-guided information dissemination (Level 1) to intensive therapist-guided treatment (Level 5) (Further details in [77]),Triple P can be delivered by a range of providers, no specialized clinical designation required - Triple P Online (TPOL) is a web-based adaptation of the Triple P intervention (Level 4), with 8 interactive online modules using videos, interactive activities, and downloadable resources, and a "dynamically generated workbook for tracking progress through the program"; completion of one module unlocks access to the next module in the sequence - Triple P Online (TPOL)- Brief is a condensed web-based adaptation of the Triple P intervention, with 5 interactive online modules using videos, interactive activities, downloadable resources, and personalized goal-setting; users are required to complete an introductory module before gaining access to the other modules, at which point they can access the rest of the modules in any order

## Supplementary Information

Below is the link to the electronic supplementary material.

Supplementary file1 (DOCX 14 KB)

## Data Availability

All data are available from the corresponding author on reasonable request.
