# Correction: Virtual Delivery of Parent Coaching Interventions in Early Childhood Mental Health: A Scoping Review

**DOI:** 10.1007/s10578-023-01632-8

**Published:** 2023-11-08

**Authors:** Catriona Hippman, Janet W. T. Mah, Megan MacFadden

**Affiliations:** BC Children’s Hospital, University of British Columbia (UBC), 4500 Oak Street, Vancouver, BC V6H 3N1 Canada

**Correction to: Child Psychiatry & Human Development**

10.1007/s10578-023-01597-8

The article “Virtual Delivery of Parent Coaching Interventions in Early Childhood Mental Health: A Scoping Review”, written by Catriona Hippman, Janet W. T. Mah, and Megan MacFadden, was originally published electronically on the publisher’s internet portal (https://link.springer.com/article/10.1007/s10578-023-01597-8) on 23 September 2023 without open access. With the author(s)’ decision to opt for Open Choice, the copyright of the article changed on 30 October 2023 to “The Author(s) 2023” and the article is forthwith distributed under the terms of the Creative Commons Attribution Section 4.1 International License (http://creativecommons.org/licenses/by/4.0/), which permits use, duplication, adaptation, distribution and reproduction in any medium or format, as long as you give appropriate credit to the original author(s) and the source, provide a link to the Creative Commons license and indicate if changes were made.

The original article has been corrected.

