# Supplementary material for: Virtual Delivery of Parent Coaching Interventions in Early Childhood Mental Health: A Scoping Review
Source: Child Psychiatry Hum Dev. 2023 Sep 23;56(3):1–37. doi: 10.1007/s10578-023-01597-8 (PMC12095452; doi:10.1007/s10578-023-01597-8)
Supplement: Supplementary file 1 — Supplementary file1 (DOCX 14 KB) [file 10578_2023_1597_MOESM1_ESM.docx]

**Title:** Virtual delivery of parent coaching interventions in early childhood mental health: A scoping review

**Journal name:** Child Psychiatry & Human Development

**Supplemental Methods: Search strings**

Search set 1:

1. “telepsychiatry”;

2. “telemental health”; and

3. “e-health AND psych* OR telehealth AND psych* OR telemedicine AND psych* OR videoconferencing AND psych* OR teleconferencing AND psych* OR videoteleconferencing AND psych*”

Search set 2:

1. “attach* AND psych* AND e-health OR attach* AND psych* AND telehealth OR attach* AND psych* AND telemedicine OR attach* AND psych* AND videoconferencing OR attach* AND psych* AND teleconferencing OR attach* AND psych* AND videoteleconferencing OR attach* AND psych* AND online OR attach* AND psych* AND virtual OR attach* AND psych* AND digital OR attach* AND psych* AND remote”;

2. “attach* AND "mental health" AND e-health OR attach* AND "mental health" AND telehealth OR attach* AND "mental health" AND telemedicine OR attach* AND "mental health" AND videoconferencing OR attach* AND "mental health" AND teleconferencing OR attach* AND "mental health" AND videoteleconferencing OR attach* AND "mental health" AND online OR attach* AND "mental health" AND virtual OR attach* AND "mental health" AND digital OR attach* AND "mental health" AND remote”;

3. “behav* AND intervention AND psych* AND e-health OR behav* AND intervention AND psych* AND telehealth OR behav* AND intervention AND psych* AND telemedicine OR behav* AND intervention AND psych* AND videoconferencing OR behav* AND intervention AND psych* AND teleconferencing OR behav* AND intervention AND psych* AND videoteleconferencing OR behav* AND intervention AND psych* AND online OR behav* AND intervention AND psych* AND virtual OR behav* AND intervention AND psych* AND digital OR behav* AND intervention AND psych* AND remote”.
